# 3D-printed skull model for enhancing training in external ventricular drainage within medical education

**DOI:** 10.1186/s41205-025-00263-0

**Published:** 2025-04-03

**Authors:** Katharina Scheidt, Fabian Kropla, Dirk Winkler, Robert Möbius, Martin Vychopen, Johannes Wach, Erdem Güresir, Ronny Grunert

**Affiliations:** 1 Department of Neurosurgery, University of Leipzig, Liebigstr. 20, 04103 Leipzig, Germany; 2 Fraunhofer-Institute for Machine Tools and Forming Technology, 02763 Zittau, Germany; 3 Biosaxony– Saxony’s Biotech, Medtech and Health Economy Cluster, Deutscher Platz 5c, 04103 Leipzig, Germany

**Keywords:** 3D-printing, External ventricular drain, Phantom, Formlabs, Elastic 50A, Neurosurgery, Surgical simulation, Training phantom, Medical education

## Abstract

**Background:**

The importance of reducing error rates in invasive procedures has led to the development of teaching phantoms. In collaboration with surgeons and engineers at the University Hospital of Leipzig, a new 3D-printed simulation model for external ventricular drainage was created. This model includes system-relevant components such as the ventricular system, the surrounding brain tissue and the skull bone to be trephined. The methodology for developing the simulation model is described in detail. Additionally, the system was initially evaluated by neurosurgeons using a Likert scale. Future studies are planned to assess the system’s accuracy and perform comparative analyses.

**Methods:**

The data required for analysis were extracted from medical images. The phantom consists of three components: the ventricular system, the brain mass, and the skull bone. The bone component was fabricated via 3D printing using a realistic hard polyamide, PA12. The ventricular system was also 3D printed as a hollow structure using a flexible material, *Elastic Resin 50 A* from *Formlabs*. The brain tissue was modeled via a cast gelatin mold. The cerebrospinal fluid was a water solution.

**Results:**

The system’s initial tests successfully simulated cerebrospinal fluid flow through the tube into the ventricular system. The skull can be trepanned. Additional materials are required at the drilling sites because of chip formation. A more pointed cannula than usual can puncture the ventricular system. With a concentration of 30 g/l, gelatin is a realistic imitation of brain tissue.

**Conclusion:**

All essential components of the skull, brain and ventricle exhibit a degree of realism that has never been achieved before. In terms of its design and reproducibility, the model is exceptionally well suited for training and consolidating methods and procedures as part of a realistic training program for the placement of external ventricular drainage.

## Background

The placement of external ventricular drainage (EVD) is an essential and life-saving procedure for neurosurgical and neurological patients to drain cerebrospinal fluid (CSF) and monitor intracranial pressure (ICP). The primary goals of EVD are to normalize elevated ICP, monitor ICP and control the microbiological situation. Increased ICP can result from various factors, including increased brain volume due to hemorrhage, swelling, or inflammation. Intracranial pressure is typically measured as CSF pressure in a sitting position. In healthy adults, the normal range is between 5 and 15 mm Hg, with the *Foramen Monroi* serving as the zero point. The auricle is used as the external reference point. Chronic ICP above 20 mm Hg can result in permanent damage and requires treatment [1]. Hydrocephalus is a medical condition characterized by abnormal enlargement of the ventricular system. If left untreated, inadequate drainage of CSF can lead to patient death. It is a frequent indication for external ventricular drainage and is commonly used in neurosurgery, emergency medicine, and intensive care. Conventional hands-free EVD procedures are often unguided and user-unfriendly, resulting in correct positioning of the catheter in only 56% of cases. In approximately 22% of procedures, the catheter is even placed incorrectly in a nonventricular CSF space [2]. The position of the catheter can be confirmed via CT scans.

### Anatomical situation

**Fig. 1:**
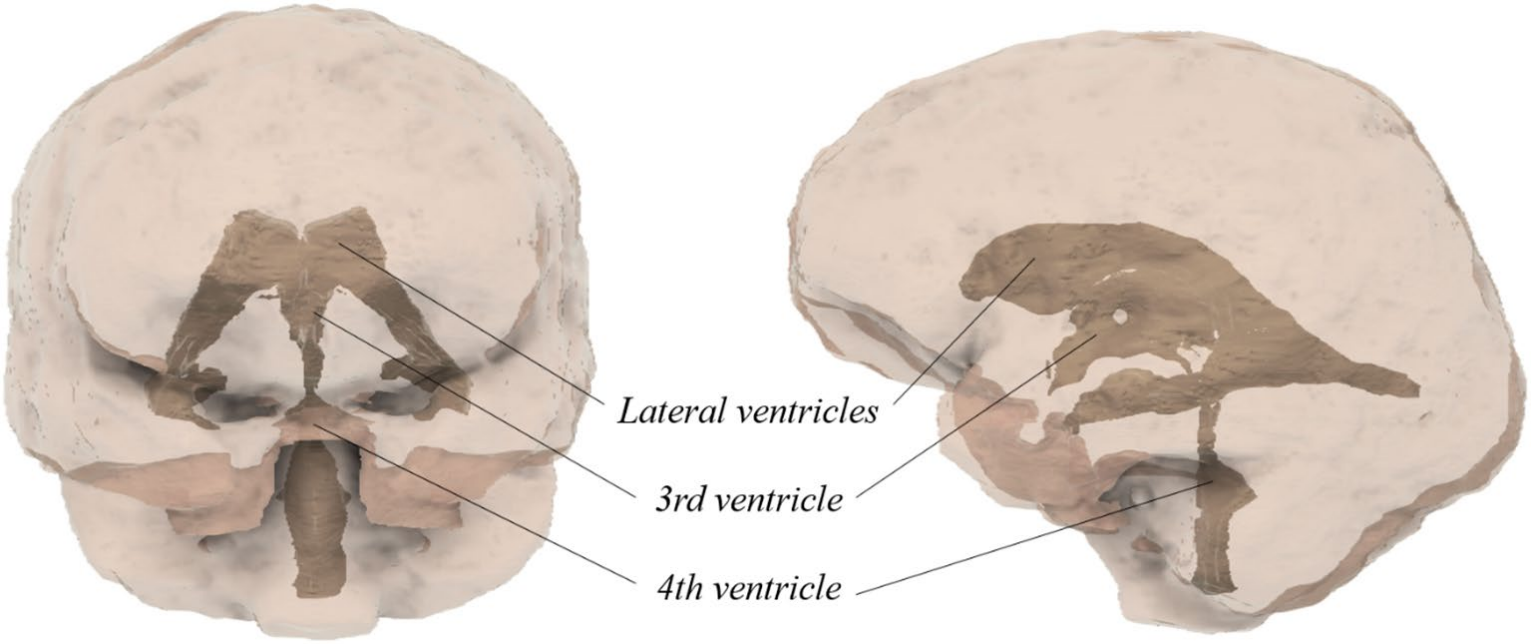
Ventricles of the brain

The ventricular system comprises two lateral ventricles (*ventriculus lateralis sinister and dexter*), each located in a cerebral hemisphere, the third ventricle (v*entriculus tertius*) situated in the diencephalon, and the fourth ventricle (v*entriculus quartus*) located in the rhomboid brain. Together with the spinal canal, they form the inner cerebrospinal fluid space. The fourth ventricle is a continuation of the central canal of the spinal cord (Fig. 1). Each ventricle contains a branched plexus of blood vessels known as the *choroid plexus*. This structure produces cerebrospinal fluid, which is then released into the ventricular system, where it contributes to the internal cerebrospinal fluid space [3]. 

The limbic system contains sensitive areas such as the thalamus, hypothalamus and hippocampus, which are located in close proximity to the ventricular system. These structures are at risk from incorrect catheter placement, which can have serious consequences. The EVD catheter is inserted through a borehole trephination and advanced into the lateral ventricles. Cerebrospinal fluid is drained through a drip chamber into a drainage bag, allowing for an estimation of intracranial pressure on the basis of the volume of fluid drained [3]. 

**Fig. 2:**
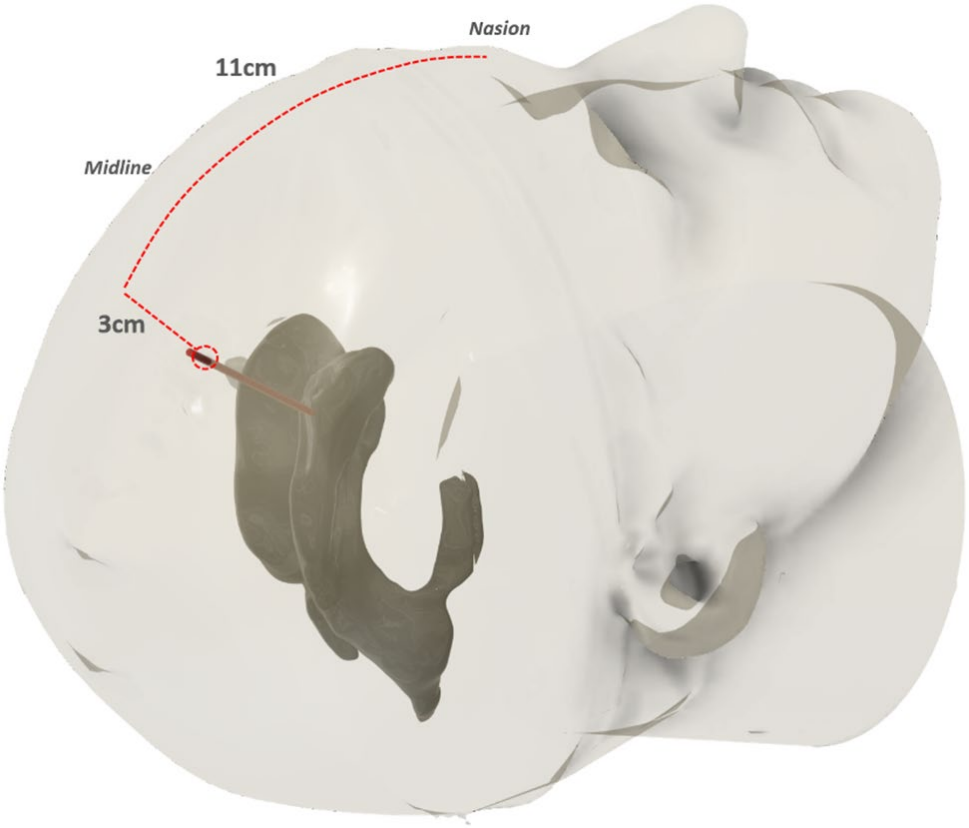
Ventricular access via Kocher’s point, with drill hole approximately 11 cm posterosuperior to the nasion and 3 cm lateral to the midline

The placement of the cannula in the ventricular system varies depending on the indication. Accurate placement requires the identification of different anatomical landmarks for trephination and the subsequent trajectory. The commonly used entry point for EVD placement is *Kocher’s point* (see Fig. 2), which is located in the frontal bone (*os frontale*), approximately 1 to 2 cm anterior to the coronal suture (*sutura coronalis*) and approximately 3 cm lateral to the midline. Another reference point is the nasal bone, which can be used to adjust *Kocher’s point* approximately 11 cm upward and 3 cm laterally [4]. 

Despite its widespread use, the error rate ranges from 4 to 40% [4]. The catheter’s position can be evaluated via a CT scan and classified according to the Kakarla system, which categorizes it into three levels: the optimal position, suboptimal position in noneloquent brain tissue, and suboptimal position in eloquent brain tissue Table 1.

**Table 1:** Grading system for catheter tip location [5]

Grade	Accuracy of placement	Location of catheter tip
1	Optimal/adequate	Ipsilateral frontal horn, including tip of third ventricle
2	Suboptimal (shallow) in noneloquent tissue	Contralateral frontal horn or lateral ventricle/corpus callosum/interhemispheric fissure
3	Suboptimal in eloquent tissue	Brainstem/cerebellum/internal capsule/basal ganglia/thalamus/occipital cortex/basal cisterns

The placement of the EVD via alternative access points such as *Keen’s Point*,* Sanchez Point*,* Frazier’s Point*, etc., was not considered in this model because of its rarity.

## Methods

The model is produced via additive manufacturing. Additive manufacturing builds components layer by layer, providing new design freedom and flexibility in construction, unlike conventional processes that remove material. The simulation model is designed for educational purposes.

### General development steps

**Fig. 3:**
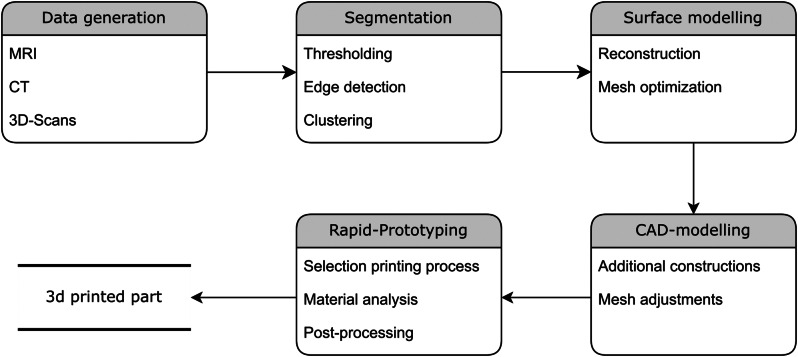
Flowchart for producing a 3D-printed model using medical data

The simulation model must be reproducible from medical image data via rapid prototyping and a reverse engineering approach. Figure 3 illustrates the procedure.

**Fig. 4:**
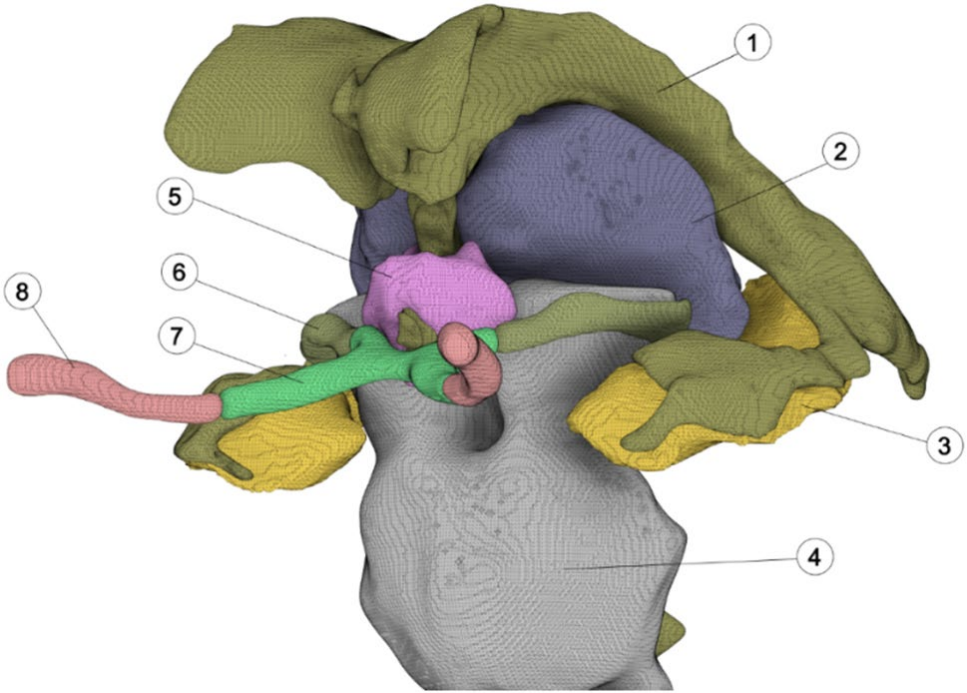
Ventricular system with surrounding structures. (1) ventricles, (2) thalamus, (3) hippocampus, (4) brainstem, (5) hypothalamus, (6) optic tract, (7) chiasm, (8) optic nerve

The design process utilizes medical data in the form of MR images, which allow the differentiation of anatomical structures and clear visualization of both hard and soft tissues. The data are recorded in DICOM format, which is the standard format for diagnostic imaging in medicine. Various common image processing techniques, such as thresholding, masking, morphological filtering, region growing, and contour detection, are used to segment the required structures. Segmenting structures from slice images enables the reconstruction of 3D models from 2D medical images. The structures were segmented via *Brainlab* software (Brainlab AG, 2024, Munich, Germany), which is widely used in clinical practice. Segmentation is performed interactively with semiautomatic methods to support navigated surgical planning. The simulation model required segmentation of the ventricular system and the surrounding areas of the limbic system (see Fig. 4).

However, polygon meshes resulting from this process may contain errors that require correction. To address these issues, *Autodesk’s Meshmixer* software (Meshmixer 3.5, Autodesk GmbH, Munich, Germany) can be used to close holes, smooth and reduce meshes, and create bridges. In the subsequent CAD modeling stage, the optimized mesh can be enhanced with additional 3D objects, such as drill holes, brackets, guides, and grooves. The modeling process was performed via *Autodesk’s Fusion 360* (version 2.0, Autodesk GmbH, Munich, Germany). Data records are uniformly transformed into the CAD program in the STL or OBJ format. The segmented structures are registered and placed in a fixed coordinate frame. Various printing processes and materials can be used for rapid prototyping as the final step in the process chain, depending on the requirements. To simulate brain tissue, a casting process using gelatin was selected.

### Design and manufacturing of the various components

The design of the individual components is based on a utility analysis, that includes the following evaluation criteria: stability, puncture resistance, toughness, durability, pressure stability, elasticity, consistency, dimensional stability and aesthetics. Different manufacturing methods were selected for producing the simulation model to meet the varying requirements of the skull model, ventricular system, and brain tissue imitation.

### Design of the ventricular system

**Fig. 5:**
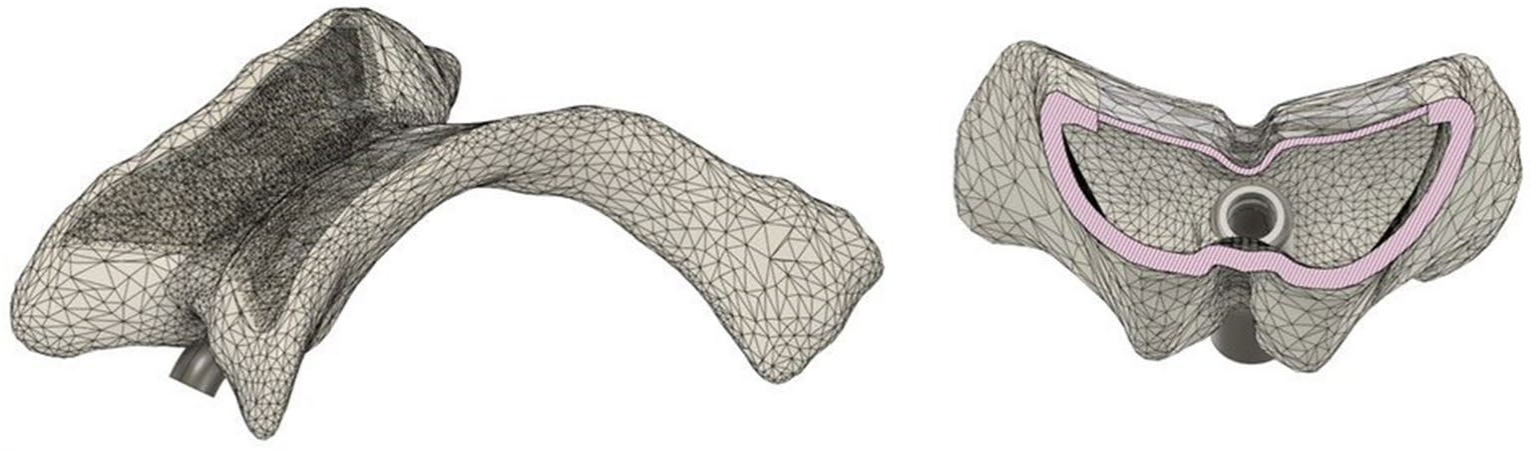
Lateral ventricles covered with a 0.5 mm membrane on the upper side. The left image shows a top view, whereas the right image shows a sectional view that highlights the differences in wall thickness

The ventricular system must meet the essential requirements of fluid absorption, leak tightness, and puncturability with cannulas. The lateral ventricles simplify the design of the ventricular system. To ensure puncturability, the system must be made of flexible material with thin walls at the puncture sites. *Elastic 50 A* was used for this purpose and printed via a *Formlabs Form 2* printer. The manufacturer specified a minimum wall thickness of 0.50 mm, which was adhered to. To ensure accuracy in 3D printing, the puncture sites at the top of the lateral ventricles were printed with a 0.50 mm layer. Additionally, the lower body was printed with a 1.00 mm layer for added stability to prevent printing errors. By adjusting the layer thicknesses during 3D printing, a ventricular system with haptic properties similar to those of the human ventricular system was created. To ensure stability during printing, the shape of the lateral ventricles was simplified. The temporal horn was omitted in this simplification, as the focus was initially on the frontal puncture. The primary focus remains on *Kocher’s point*, which is unaffected by these modifications. A tube connection between the two lateral ventricles simulates cerebrospinal fluid flow. Figure 5 shows the modified ventricles with a membrane over the puncture site, positioned above *Kocher’s point.*

### Manufacturing of the ventricular system

**Fig. 6:**
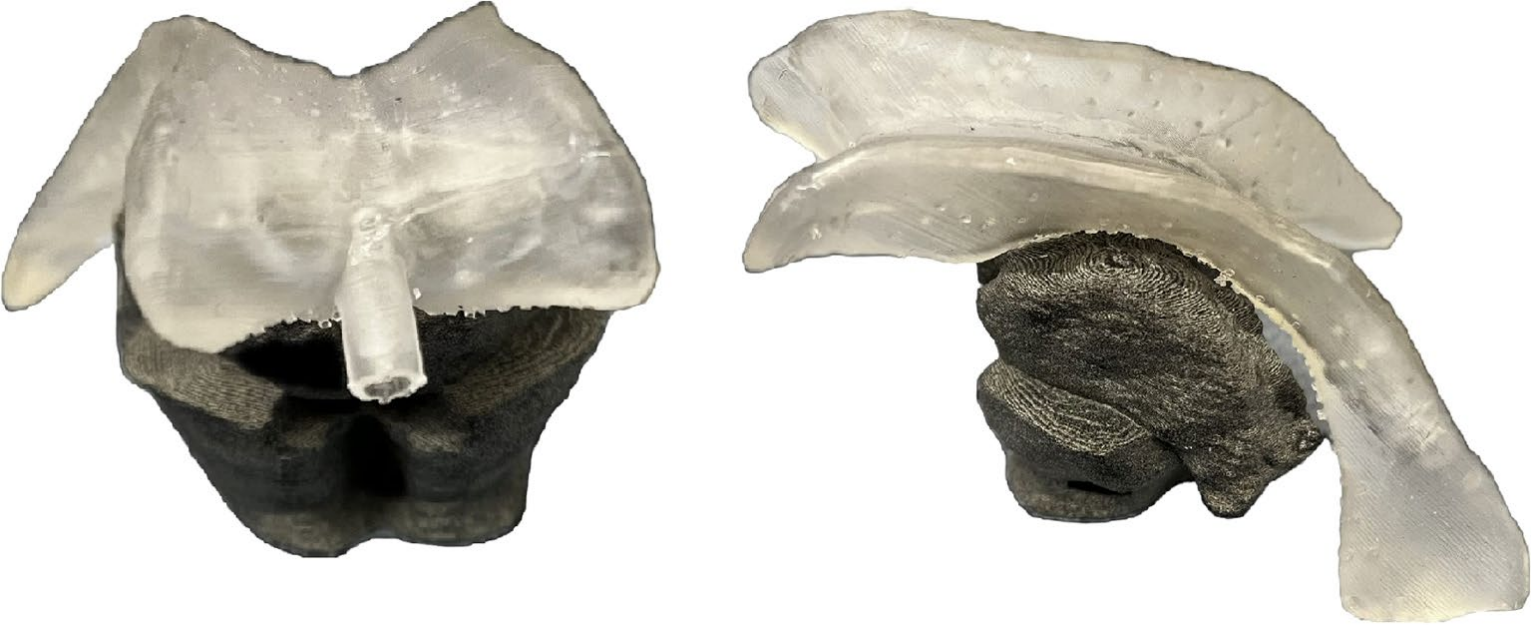
Lateral ventricles, modified and produced via 3D printing technology, made of elastic 50 A resin

An elastic material was utilized to print the ventricular system using the Formlabs Form 2 printer (Formlabs GmbH, Berlin, Germany). This printer employs the stereolithography (SLA) process, which is a subset of the broader category of Vat Photopolymerization additive manufacturing technologies. This process involves curing light-sensitive plastic base monomers layer by layer using a laser unit on a platform. It allows for layer thicknesses ranging from 0.05 mm to 0.25 mm. Postprocessing is needed, which includes cleaning the components with alcohol and curing them with UV light. The ventricular system was created using the soft material Elastic 50 A. It features the desired cavity and can be filled with liquid (Fig. 6).

### Design and manufacturing of the brain mass

**Fig. 7:**
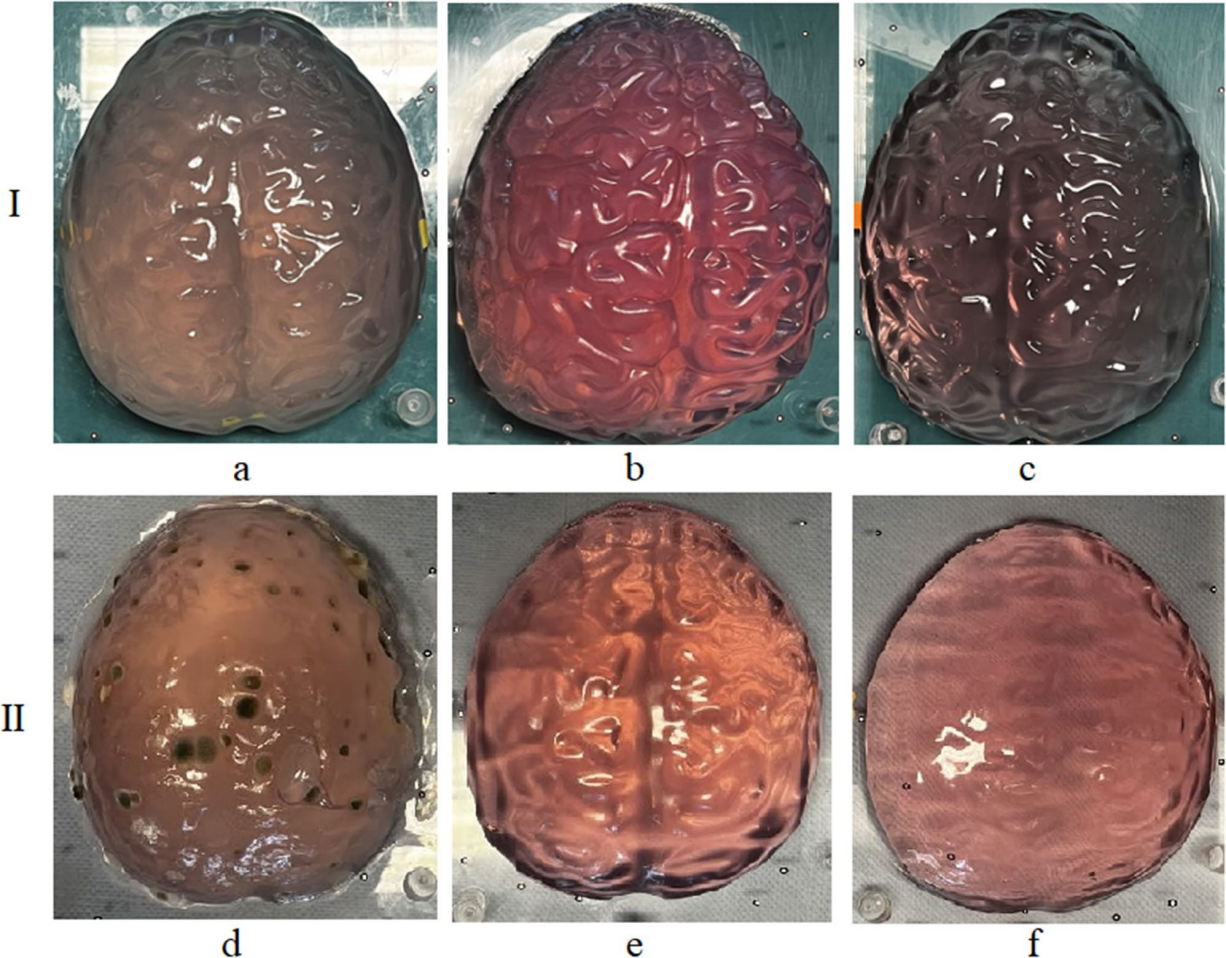
Brain mass phantoms made from gelatin: I: phantoms on day 1; II: phantoms on day 7; **a**,**d**: gelatin with H_2_O; **b**, **e**: gelatin with H_2_O (50%), isopropanol (25%) and glycerol (25%); c, f: gelatin with citric acid (5 g/l) and H_2_O

To achieve a realistic representation of the simulation model, it was necessary to simulate the brain tissue. However, determining the material properties of brain matter is challenging because of its complex biological conditions. Loosemann [6] investigated the use of gelatin as an imitation of brain matter and reported that a concentration of 29.2 g/l closely mimics the consistency of brain tissue. Durability and consistency are prioritized over design when simulating brain tissue. Since then gelatin has deteriorated rapidly. Therefore, we searched for appropriate preservatives to enhance the durability of the model. Isopropanol, combined with glycerin and citric acid, was tested as a preservative. The molding process was evaluated daily via the mold rating scale. For the initial tests, gelatin was poured into a plastic mold with simulated brain convolutions and then hardened in a refrigerator. Figure 7 shows the gelatin phantoms on the first and seventh days after casting.

**Fig. 8:**
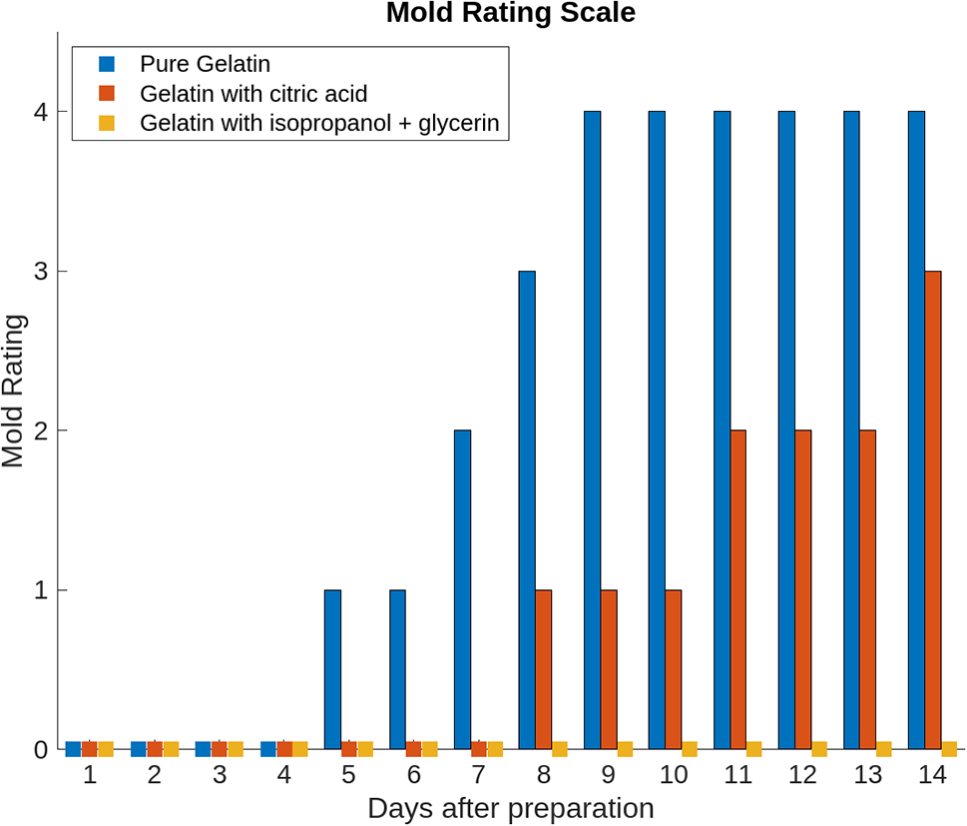
Mold rating scale for the assessment of mold formation on gelatin phantoms. The rating scale ranged from 0 to 4, where 0 indicated that the samples were completely free of mold growth and 4 indicated that the samples were completely covered with mold

Figure 7 shows that the pure gelatin phantom exhibited significant mold growth by the seventh day after casting. The mold has spread extensively over the phantom, and liquid films have formed on its surface. The phantom containing citric acid has lost its contours but shows no mold growth. Only the phantom containing isopropanol and glycerin remains in its original state, with the contours of the cerebral convolutions still visible. Mold growth was evaluated over a 14-day period via the mold rating scale, as illustrated in Fig. 8.

The only stable phantom within the 14-day time frame was the one containing isopropanol and glycerin. The other two phantoms develop mold and liquid films after 5 and 8 days, respectively (see Fig. 8). After five days, pure gelatin was unsuitable for EVD modeling. Adding citric acid extends usability to nine days, but this shelf life remains too short for the intended purpose. On the basis of these data, 500 ml of water, 250 ml of isopropanol, and 250 ml of glycerin were added to 29.2 g/l gelatin for all subsequent phantoms.

### Design of the skull bone

**Fig. 9:**
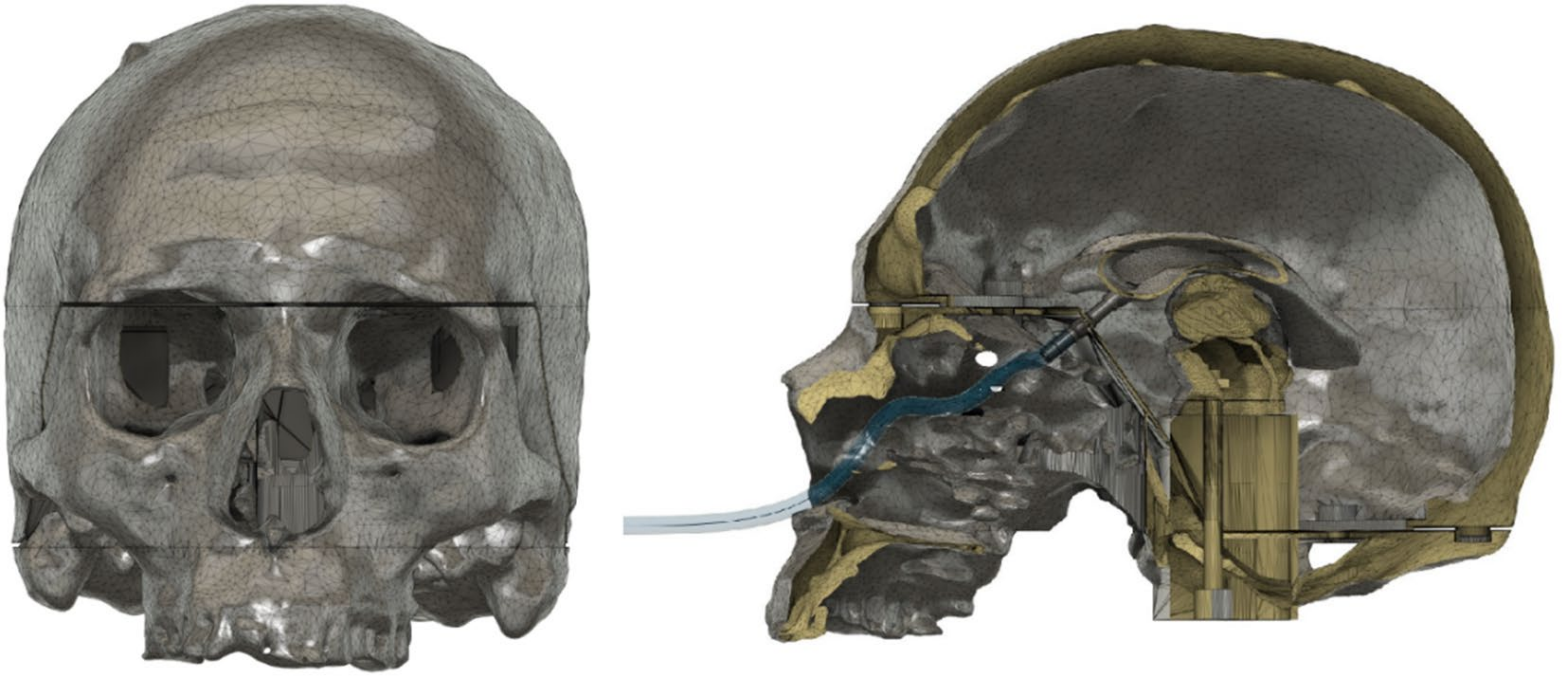
Front view of the skull model on the left and sectional view on the right. The ventricular system is connected to the brainstem with the thalamus, making it an exchange element

The head model is designed to securely hold the brain mass and ventricular system, and is based on CT scans. It must have sufficient density to enable the gelatin to mold around the ventricular system and be supported by the skull. Figure 9 illustrates the holder for the upper brain stem, including the thalamus, within the skull. The ventricular system, consisting of two modified lateral ventricles, is attached to it. Grooves for square nuts are incorporated into the lower part of the brainstem on the ventricle. These components can be secured in place by inserting them from the underside of the model through the brainstem. The ventricular system is connected to a tube that allows cerebrospinal fluid to enter the ventricles (see Fig. 9). This connection is made through the nasal cavity. Once the ventricular system is inserted, the two halves of the skull model can be closed and secured with screws and nuts.

### Manufacturing of the skull bone

The skull phantom was produced using the Multi Jet Fusion (MJF) process (Hewlett-Packard (HP) 2016, Palo Alto, USA). This process, which falls under the Powder Bed Fusion category, involves melting powder to shape it into a component. In the MJF process developed by Hewlett-Packard, a fusion agent is applied to the top layer of powder and then irradiated with an infrared energy source, causing the powder to fuse. A detailing agent, which inhibits heat, is applied to the structures around the contours. This enables the production of high-precision edges. The skull model was created via polyamide 12 (PA12), a plastic known for its high mechanical, thermal, and chemical resistance and Shore Hardness of 80D. The Shore hardness ensures precise replication of the bone and allows for accurate verification of the ventricular drainage position on CT scans.

## Results

**Fig. 10:**
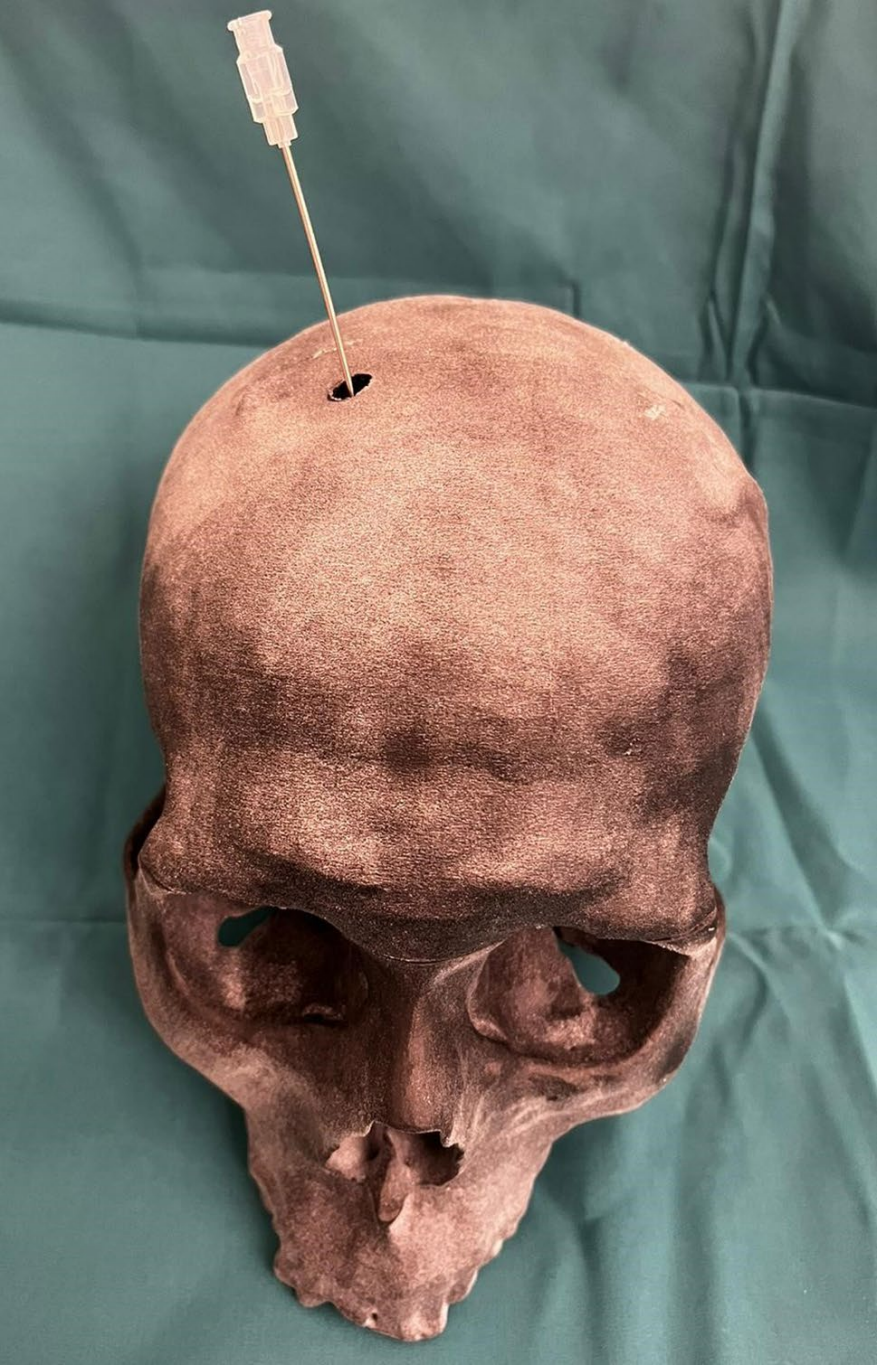
Skull model with cannula guidance through Kocher’s point

The phantom model consists of two shells, allowing for the replacement of the ventricular system without discarding the entire model in the event of a puncture. Once the ventricular system is inserted, the two skull shells should be secured together via screws through the designated holes. The cut surfaces of the shells are then sealed with modeled plates and silicone. To pour the gelatin, a trephination drill hole is made at *Kocher’s point*, and the gelatin is poured through this hole, which will later be used for the puncture. Figure 10 shows the skull phantom and the cannula access via *Kocher’s point.* The simulation model was reviewed by participating and experienced neurosurgeons, who confirmed the realism of its components and the feasibility of its application.

As part of the initial test series, the system was evaluated using a Likert scale. The study included two consultants, two specialists, and three residents, all specializing in neurosurgery. The primary objective was to assess the system and specific aspects of the experimental setup, identifying and addressing potential weaknesses prior to conducting accuracy studies. Responses to the eight-question survey were rated on a scale from 1 (strongly disagree) to 5 (strongly agree), and the mean scores from the seven participants were calculated. The results are summarized in Table 2.

**Table 2:**
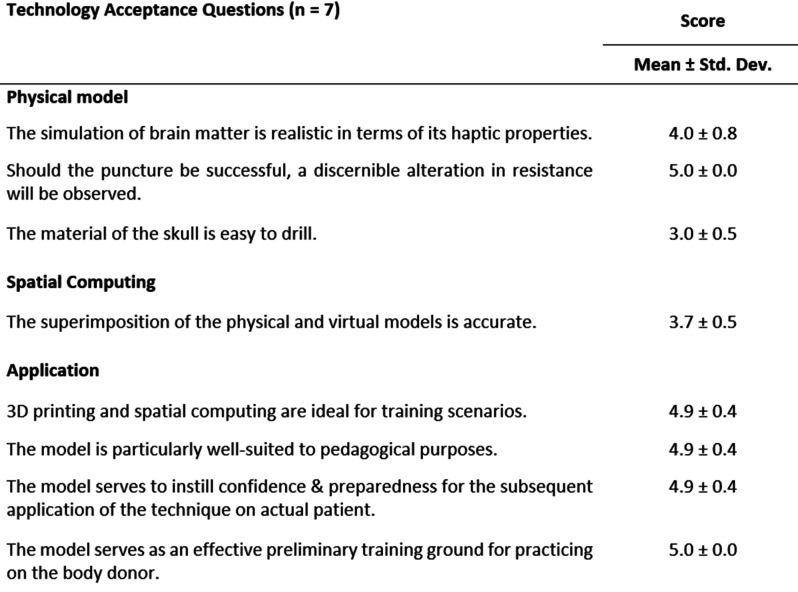
The initial study involving two consultants, two specialists, and three residents, all specializing in neurosurgery. Rating coded on a likert scale: 1, strongly disagree; 2, disagree; 3, neutral; 4, agree; 5, strongly agree

The study demonstrates a high level of acceptance and strong support for the application of 3D printing technology in conjunction with spatial computing in medical education. Notably, during the ventricular system puncture, all participants reported perceiving resistance, which functioned as haptic feedback indicative of a successful puncture. However, the evaluation also revealed several limitations in the model. Specifically, the material employed to simulate the skull bone was found to exhibit insufficient drillability, with participants noting a tendency for the material to deform or twist under drilling forces. Furthermore, the precision of the overlay between the virtual and physical models was deemed inadequate, highlighting a need for improved alignment accuracy.

## Discussion

The feasibility of training and simulation models for external ventricular drainage is underscored by the successful use of 3D printing for the skull and ventricular system, as well as the realistic simulation of brain tissue with the gelatin phantom. The skull model was manufactured successfully via MJF technology, which serves as the structural core of the model, allowing for precise drill holes as entry points for ventricular catheters along the intended trajectories. The material used, PA12, accurately replicates the hardness of bone tissue. Future development could aim to mimic the porous internal structure of bone. To address this, two approaches will be evaluated. The first involves adjusting the wall thickness at the locations designated for trepanation on the model. A thicker wall layer retains more loose powder, thereby increasing resistance during drilling, while a thinner layer reduces the loose powder volume, resulting in lower resistance. The second approach entails testing various lattice structures between the walls, as these configurations significantly impact the mechanical properties and density. The goal is to determine whether one of these variants can accurately replicate the porosity of bone. A limitation of the current skull model, in terms of sustainability, is that repeated drilling cannot be performed without replacing the upper shell. This issue will be addressed in future research phases by incorporating interchangeable plates at the designated insertion points. CT scans effectively image the PA12 material, enabling long-term tracking of catheter placement by correlating prominent points from patient data (MRI or CT) with the catheter’s position in the model.

The ventricular system has been printed successfully. Simulating the shape of the ventricular system is feasible using flexible materials. However, the research group was unable to replicate the haptic properties with available 3D printing materials in a way that would enable the use of the Spielberg catheter. An alternative option is to use spinal needles, which have various diameters and length. A suitable substitute would be a cannula with a diameter of 0.9 mm and a length of 120 mm. The resulting hole in the flexible ventricular system material is sufficiently small to contract after creation. The filling volume of the ventricular system is precisely calibrated, allowing for refilling after trephination with minimal pressure while maintaining system integrity. As a result, the ventricular system does not need to be replaced after each puncture.

Although 3D printing has made significant advancements, it remains limited, particularly when elastic materials are used, which are more challenging to print. The ventricular system in this study was printed via *Elastic 50 A* resin from *Formlabs*. In late 2023, *Formlabs* introduced a silicone material that will be tested as an alternative in the next phase of development.

The brain tissue was replicated using gelatin, which can be preserved for several weeks when combined with a solution of isopropanol and glycerin. However, brain tissue may shrink over time because of the evaporation of water-based gelatin. Despite this, the subsequent filling of the models with gelatin was successful. Further tests are to be conducted with a variety of materials, including soft rubber silicones, to ascertain their long-term mold stability.

## Conclusion

Additive manufacturing opens new possibilities for creating simulation models in medical education. These models can be customized to fit specific procedures and produced cost-effectively. With a diverse range of materials available, it is possible to replicate biological tissues with varying degrees of hardness and tactile feedback. In the future, the model will also be used to evaluate a spatial computing-based system for catheter placement in the brain, facilitating a comparative study between freehand EVD and guided EVD.

## Data Availability

No datasets were generated or analysed during the current study.
